# Basal forebrain atrophy in frontotemporal dementia

**DOI:** 10.1016/j.nicl.2020.102210

**Published:** 2020-02-13

**Authors:** Rhian S. Convery, Mollie R. Neason, David M. Cash, M. Jorge Cardoso, Marc Modat, Sebastien Ourselin, Jason D. Warren, Jonathan D. Rohrer, Martina Bocchetta

**Affiliations:** a Dementia Research Centre, Department of Neurodegenerative Disease, UCL Queen Square Institute of Neurology, University College London, London, United Kingdom; b Centre for Medical Image Computing, Department of Medical Physics and Biomedical Engineering, University College London, London, United Kingdom; c School of Biomedical Engineering and Imaging Sciences, King's College London, London, United Kingdom

**Keywords:** Frontotemporal dementia, MRI, Basal forebrain, Volumetry

## Abstract

**Background:**

The basal forebrain is a subcortical structure that plays an important role in learning, attention, and memory. Despite the known subcortical involvement in frontotemporal dementia (FTD), there is little research into the role of the basal forebrain in this disease. We aimed to investigate differences in basal forebrain volumes between clinical, genetic, and pathological diagnoses of FTD.

**Methods:**

356 patients with FTD were recruited from the UCL Dementia Research Centre and matched on age and gender with 83 cognitively normal controls. All subjects had a T1-weighted MR scan suitable for analysis. Basal forebrain volumes were calculated using the Geodesic Information Flow (GIF) parcellation method and were compared between clinical (148 bvFTD, 82 svPPA, 103 nfvPPA, 14 PPA–NOS, 9 FTD–MND), genetic (24 *MAPT,* 15 *GRN,* 26 *C9orf72*) and pathological groups (28 tau, 3 FUS, 35 TDP-43) and controls. A subanalysis was also performed comparing pathological subgroups of tau (11 Pick's disease, 6 FTDP-17, 7 CBD, 4 PSP) and TDP-43 (12 type A, 2 type B, 21 type C).

**Results:**

All clinical subtypes of FTD showed significantly smaller volumes than controls (*p* ≤ 0.010, ANCOVA), with svPPA (10% volumetric difference) and bvFTD (9%) displaying the smallest volumes. Reduced basal forebrain volumes were also seen in *MAPT* mutations (18%, *p* < 0.0005) and in individuals with pathologically confirmed FTDP-17 (17%), Pick's disease (12%), and TDP-43 type C (8%) (*p* < 0.001).

**Conclusion:**

Involvement of the basal forebrain is a common feature in FTD, although the extent of volume reduction differs between clinical, genetic, and pathological diagnoses. Tauopathies, particularly those with *MAPT* mutations, had the smallest volumes. However, atrophy was also seen in those with TDP-43 type C pathology (most of whom have svPPA clinically). This suggests that the basal forebrain is vulnerable to multiple types of FTD-associated protein inclusions.

## 1 Introduction

Frontotemporal dementia (FTD) is a clinically, genetically, and pathologically heterogeneous disease. FTD can be characterised clinically by personality change and cognitive dysfunction, known as behavioural variant FTD (bvFTD) (Rascovsky et al., 2011) or by language difficulties, termed primary progressive aphasia (PPA) (Gorno-Tempini, 2011). In around a third of cases, FTD is caused by a genetic mutation, usually in one of three genes: microtubule associated protein tau (*MAPT*), chromosome 9 open reading frame 72 (*C9orf72)* or progranulin (*GRN*) (Rohrer and Warren, 2011). Neuropathologically, tau, transactive response DNA binding protein 43 kDa (TDP-43), and fused in sarcoma (FUS) inclusions are the most common cause of brain abnormalities in FTD (Lashley et al., 2015; Mackenzie et al., 2009). FTD is classically associated with a pattern of atrophy centred around the frontal and temporal lobes, however, a number of studies have also shown early subcortical involvement (Rohrer et al., 2010; Seeley et al., 2008; Whitwell et al., 2012).

**Fig. 1:**
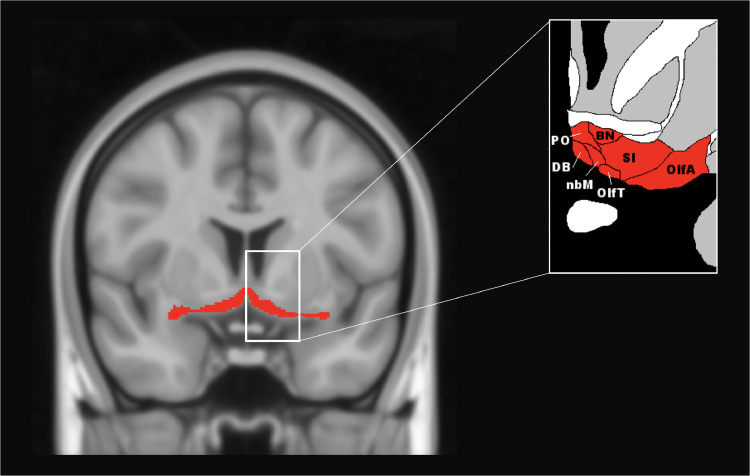
Representative figure of the basal forebrain and its anatomy, based on Ding et al. (2016). The basal forebrain segmentation is mapped on to the T1-weighted ICBM152 2009c nonlinear symmetric - 1 × 1 × 1 mm template (McConnell Brain Imaging Centre, Montreal Neurological Institute, McGill University). PO = preoptic area, BN = bed nucleus, DB = nucleus of the diagonal band, nbM = basal nucleus of Meynert, OlfA = olfactory area, OlfT = olfactory tract, SI = substantia innominata. The coronal slice corresponds to MNI *y* = 131.

The basal forebrain is a subcortical structure located on the medio-ventral portion of the brain, ventral to the striatum (Fig. 1). It consists of a number of different structures, including the medial septal nucleus, the diagonal band of Broca, and the nucleus basalis of Meynert (Mesulam, 2004a), with the major cholinergic pathways arising from these nuclei and innervating large portions of the neocortex and limbic system (Zaborszky et al., 2015). Cholinergic activity linked to the basal forebrain has been shown to be crucial for several cognitive processes including attention (Baxter and Chiba, 1999; Bracco et al., 2014; Muir et al., 1996) learning, and memory (Sarter et al., 2003).

To date, the vast majority of research into the basal forebrain has been carried out in Alzheimer's disease (AD). Structural MRI (Teipel et al., 2005) and post-mortem studies (Lehéricy et al., 1993; Nagai et al., 1983; Vogels et al., 1990) have shown severe degeneration of the basal forebrain cholinergic nuclei in AD the extent of which is associated with the severity of cognitive impairment (Bierer et al., 2002; Mesulam, 2013; Mesulam, 2004b; Perry et al., 1977). So far, structural MRI studies of the basal forebrain in FTD have shown a reduction in volume in PPA (Schaeverbeke et al., 2017; Teipel et al., 2016, 2014) and suggested that basal forebrain nuclei are crucial for language function (Simić et al., 1999; Teipel et al., 2014). Research in FTD-associated tauopathies, including progressive supranuclear palsy (PSP) (Kasashima and Oda, 2003; Tagliavini et al., 1984) and corticobasal degeneration (CBD) (Kasashima and Oda, 2003) has also shown a reduction in basal forebrain volumes at post-mortem. However, comprehensive research into basal forebrain involvement in other forms of FTD is limited. The primary objective of this study was to investigate the pattern of basal forebrain involvement in the different clinical, genetic, and pathological forms of FTD.

## 2 Methods

### 2.1 Participants

**Fig. 2:**
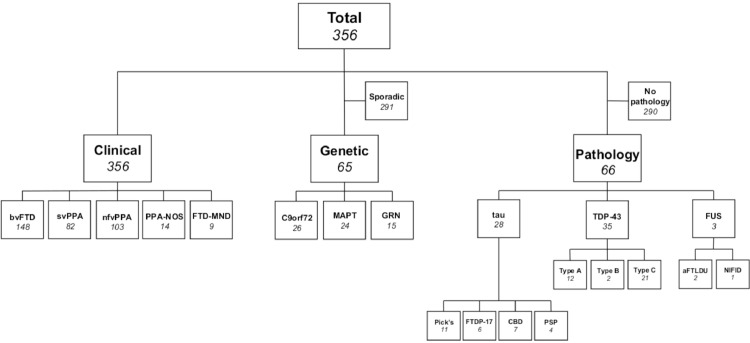
Overview of the study cohort, showing how patients were stratified in to clinical, genetic, and pathological groups and further divided in to pathological subtypes.

We identified 356 patients from the UCL Dementia Research Centre FTD cohort who had a good quality T1-weighted magnetic resonance (MR) scan. Of these patients 148 were diagnosed with bvFTD, 82 with semantic variant PPA (svPPA), 103 with nonfluent variant PPA (nfvPPA), 14 with PPA not otherwise specified (PPA–NOS), and 9 with a diagnosis of FTD with motor neurone disease (FTD–MND), see Fig. 2. We did not include patients with logopenic variant PPA who are likely to have underlying AD pathology. These patients were matched on age and gender with 83 cognitively normal controls who also had a suitable T1–weighted MR scan that passed standard quality control checking. Informed consent was obtained from all participants, and the study was approved by the local ethics committee.

### 2.2 MR acquisition

All participants had a 3D T1-weighted MR scan on one of three scanners: 215 on a 1.5T GE MRI scanner, 176 on a 3T Siemens Trio MRI scanner, and 48 on a 3T Siemens Prisma MRI scanner. MR scans underwent standard methods for quality control and were excluded if scans showed artefacts, motion, any vascular lesions or any other brain lesions not related to FTD.

### 2.3 Patient groups

We also stratified participants by their genetic and pathological cause of FTD. Within the FTD patient group, 65 patients were carriers of a mutation in one of the FTD causing genes: 24 in the *MAPT* (Ghetti et al., 2015), 15 in the *GRN* (Baker et al., 2006; Cruts et al., 2006), and 26 in the *C9orf72* (DeJesus-Hernandez et al., 2011; Renton et al., 2011) group (see Supplementary Table 1 for the individual mutations). Post-mortem pathological confirmation was available for 66 FTD patients. Neuropathological examination of brain tissue was carried out at the Queen Square Brain Bank for Neurological Disorders at UCL following standard histopathological methods (see Supplementary Material) where underlying pathology was identified for these patients: 28 with a tauopathy, 35 with a TDP-43 proteinopathy and 3 FUSopathy. None of the patients had significant secondary pathology. We also performed a secondary analysis to investigate whether the specific type of pathology had an impact on basal forebrain volumes: in the tauopathies, 11 with Pick's disease, 7 with CBD, 4 with PSP and 6 with FTDP-17 (i.e. pathology associated with *MAPT* mutations); and in the TDP-43 proteinopathies, 12 with type A, 2 with type B, and 21 with type C). In the FUS pathology group, there were 2 patients with atypical frontotemporal lobar degeneration (FTLD) with ubiquitin inclusions (aFTLDU) and 1 with neuronal intermediate filament inclusion disease (NIFID) so no subanalysis was performed.

### 2.4. Statistical analysis

Basal forebrain volumes were automatically extracted using the Geodesic Information Flow algorithm (GIF) (Cardoso et al., 2015). GIF is an automated technique for the segmentation and volume extraction of 162 grey matter and white matter regions on 3D T1-weighted MR images. It is a multi-atlas propagation approach, which uses the neuroanatomical annotations from the Neuromorphometric atlas [https://mindboggle.info/braincolor/] to parcellate in a step-wise way MR images in their native space, after bias-correction of field inhomogeneities. We focused on comparing the whole basal forebrain structure, calculated by summing left and right basal forebrain volumes. The basal forebrain volume was expressed as a percentage of total intracranial volume (TIV), computed with statistical parametric mapping (SPM) version 12 software (www.fil.ion.ucl.ac.uk/spm/) in MATLAB (Malone et al., 2015). All segmentations underwent quality control checking. Statistical analyses were performed in SPSS software (SPSS Inc, Chicago, IL, USA) v22.0, between the patient groups and controls, tested using an ANCOVA and adjusting for scanner type, gender, age, and disease duration (for patient group comparisons). Results were corrected for multiple comparisons (Bonferroni's correction) with a threshold of *p* < 0.01.

## 3 Results

Demographic data as well as basal forebrain volumes for FTD patient groups and controls are reported in Table 1**.** The mean disease duration for the FTD group, at the time of scan, was 3.8 years (SD = 3.1). There was no significant difference in age between FTD patients (64.2 years, SD = 8.4) and controls (61.5 years, SD = 11.8, *p* = 0.454, *t*-test), and there was no difference in scanner type (*p* = 0.151, Chi-square test), or gender (*p* = 0.349, Chi-square test). No significant differences in scanner type were found across clinical, genetic, and pathological diagnoses (*p* = 0.175, *p* = 0.418, *p* = 0.604, Chi-square test). There was also no significant difference in disease duration across clinical and pathological diagnoses (*p* = 0.057, *p* = 0.556, ANOVA). However, compared to *C9orf72* (5.5 years) and *MAPT* (5.7 years), *GRN* carriers did have a significantly shorter disease duration (2.9 years, *p* = 0.016).

**Table 1:** Demographics and clinical variables for the FTD patient groups and controls, together with basal forebrain volumes. Values denote means (standard deviation) or n (%). Mean volumes in bold denote significant *p*-values in comparison to controls once adjusting for multiple comparisons.

Groups		n	Gender, male	Age at scan (years)	Disease duration (years)	Basal forebrain volume as % of TIV	*p*-value
**Controls**		83	45 (54%)	63.4 (10.8)	–	0.071 (0.007)	–
**Clinical**	**bvFTD**	148	103 (70%)	61.5 (8.2)	5.1 3.3)	**0.066** (0.008)	<0.0005
	**nfvPPA**	103	49 (48%)	68.4 (8.5)	4.3 (2.2)	**0.068** (0.007)	<0.0005
	**svPPA**	82	46 (56%)	63.8 (7.3)	4.8 (2.5)	**0.065** (0.007)	<0.0005
	**PPA-NOS**	14	10 (71%)	63.9 (6.2)	3.3 (1.7)	**0.067** (0.006)	0.005
	**FTD–MND**	9	5 (56%)	65.3 (4.1)	4.5 (2.4)	**0.066** (0.007)	0.010
**Genetic**	***C9orf72***	26	17 (65%)	61.6 (7.0)	5.5 (3.3)	0.072 (0.007)	0.857
	***GRN***	15	7 (47%)	62.6 (6.7)	2.9 (2.7)	0.069 (0.006)	0.107
	***MAPT***	24	15 (63%)	55.4 (7.0)	5.7 (3.2)	**0.059** (0.008)	<0.0005
**Pathological**	**TDP-43**	35	21 (60%)	63.3 (7.9)	4.3 (2.5)	**0.068** (0.006)	0.003
	**TDP-A**	12	6 (50%)	61.6 (8.2)	3.1 (1.6)	0.069 (0.005)	0.133
	**TDP-B**	2	1 (50%)	55.0 (9.4)	5.0 (3.7)	0.077 (0.009)	0.403
	**TDP-C**	21	14 (67%)	65.1 (7.3)	4.9 (2.7)	**0.066** (0.005)	0.001
	**Tau**	28	21 (72%)	61.8 (9.7)	4.7 (2.3)	**0.065** (0.007)	<0.0005
	**Pick's disease**	11	8 (73%)	61.3 (2.5)	4.2 (2.0)	**0.063** (0.006)	<0.0005
	**FTDP-17**	6	4 (67%)	52.4 (6.2)	5.8 (3.1)	**0.060** (0.009)	<0.0005
	**CBD**	7	5 (71%)	59.5 (9.0)	4.5 (0.9)	0.071 (0.006)	0.609
	**PSP**	4	4 (100%)	76.9 (7.3)	5.1 (3.9)	0.064 (0.003)	0.030
	**FUS**	3	2 (67%)	43.9 (13.6)	3.3 (2.1)	0.066 (0.008)	0.172

**Fig. 3:**
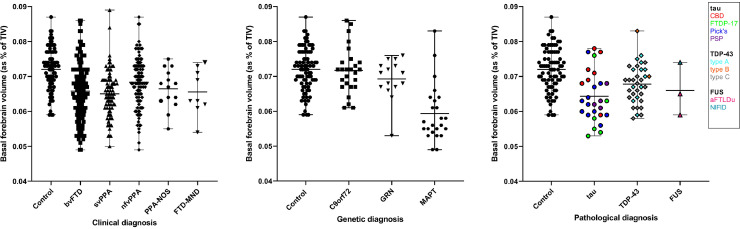
Volume of the basal forebrain, as a percentage of total intracranial volume, between FTD patients and controls, by clinical, genetic, and pathological diagnosis.

Basal forebrain volumetric differences between controls and clinical, genetic, and pathological groups are displayed in Fig. 3. Overall, FTD patients had significantly smaller basal forebrain volumes than controls (8% difference overall, *p* < 0.0005, ANCOVA) regardless of the clinical, genetic, or pathological diagnosis. All individual clinical subtypes showed significantly smaller volumes than controls (*p* ≤ 0.010, ANCOVA). The svPPA group had the smallest volumes (10% difference from controls), followed by bvFTD (9%), FTD–MND (9%), PPA–NOS (8%), and lastly nfvPPA (5%). Both the bvFTD (4% difference, *p* = 0.016) and svPPA (5%, *p* = 0.003) groups had reduced basal forebrain volumes in comparison to nfvPPA patients (Table 2). No other significant differences were found when comparing within clinical diagnosis.

**Table 2:** Basal forebrain volumetric comparisons between controls and the different clinical, genetic, and pathological groups. Volumes are adjusted for age, scanner type and gender, as well as disease duration for within patient group comparisons.

**Clinical**	**FTD–MND**		**bvFTD**		**nfvPPA**		**PPA-NOS**		**svPPA**	
	% difference	*p*-value	% difference	*p*-value	% difference	*p*-value	% difference	*p*-value	% difference	*p*-value
**Controls**	9%	0.010	9%	<0.0005	5%	<0.0005	8%	0.005	10%	<0.0005
**FTD–MND**			0%	0.911	−4%	0.293	−1%	0.847	1%	0.807
**bvFTD**					−4%	0.016	−1%	0.876	1%	0.373
**nfvPPA**							3%	0.325	5%	0.003
**PPA-NOS**									2%	0.565
**Genetic**	***C9orf72***		***MAPT***		***GRN***					
	% difference	*p*-value	% difference	*p*-value	% difference	*p*-value				
**Controls**	0%	0.857	18%	<0.0005	4%	0.107				
***C9orf72***			17%	<0.0005	3%	0.210				
***MAPT***					14%	<0.0005				
**Pathological**	**Tau**		**FUS**		**TDP-43**					
	% difference	*p*-value	% difference	*p*-value	% difference	*p*- value				
**Controls**	10%	<0.0005	10%	0.172	6%	0.003				
**Tau**			−2%	0.730	5%	0.092				
**FUS**					3%	0.729				

When stratifying by genetic diagnosis, only the *MAPT* group had significantly smaller basal forebrain volumes compared with controls (18% difference, *p* < 0.0005) (Tables 1 and 2). Patients in the *MAPT* group also displayed reduced volumes compared to both the *C9orf72* (17%, *p* < 0.0005), and *GRN* (14%, *p* < 0.0005) groups. No volumetric differences in basal forebrain volumes were found between the *C9orf72* and *GRN* genetic groups.

When stratifying by pathology, the smallest volumes were seen in the tauopathies (10% difference from controls, *p* < 0.0005). However, there was also a significant difference from controls for the TDP-43 proteinopathies (6% difference, *p* = 0.003), with a non-significant difference in the FUSopathies (10% difference, *p* = 0.172). Within the specific tauopathies, we found that only subjects with FTDP-17 and Pick's disease (17% and 12%, *p* < 0.0005) had smaller volumes than controls, although there was a trend to lower volumes in those with PSP (*p* = 0.030). In the TDP-43 proteinopathies, only those with type C had significantly smaller basal forebrain volumes (8%, *p* = 0.001) than controls.

## 4 Discussion

Using the robust GIF parcellation method, this study has demonstrated that the basal forebrain shows significant volume reduction in FTD. Three major findings emerged from this study. First, the basal forebrain is reduced across the different clinical presentations of FTD. Second, individuals with *MAPT* mutations have significantly smaller basal forebrain volumes than the other genetic groups. Thirdly, tauopathies have the smallest basal forebrain volumes, driven by both patients with FTDP-17 (i.e. *MAPT* mutations) and Pick's disease, whilst in the TDP-43 proteinopathies, lower volumes are driven by a decrease in those with type C pathology.

We show similar reductions of basal forebrain volumes in all clinical diagnoses of FTD within the study. Individuals with svPPA and bvFTD had the lowest volumes, with lower basal forebrain volumes in comparison to nfvPPA in both of these groups. This finding is in line with previous studies showing significant reductions in basal forebrain volumes particularly in svPPA, and to a lesser extent in nfvPPA (Schaeverbeke et al., 2017; Teipel et al., 2016). However, to our knowledge this is the first study to demonstrate the differences in basal forebrain volumes across all of the FTD diagnoses, including bvFTD.

Our results also indicate that, within the genetic and pathological diagnoses, the basal forebrain is mainly involved in individuals with tauopathies, specifically those with *MAPT* mutations (FTDP-17), and also those with Pick's disease pathologically. Previous research has demonstrated the role of tau in basal forebrain degeneration. Early deposition of tau in the basal forebrain is seen in AD, which correlates with cognitive decline (Vana et al., 2011). Other research has suggested that tau pathology in the basal forebrain is an early event in the transition from mild cognitive impairment to AD (Mesulam, 2004a). Although FTD-associated tau differs from the tau neurofibrillary tangle pathology seen in AD, it appears that different structural conformations of tau can similarly affect the basal forebrain. The fact that different types of FTD-associated tau, including FTDP-17 and Pick's disease, both exhibited reduced basal forebrain volumes, supports this notion. Research has demonstrated that cholinergic nuclei are differently affected by pathogenetic mechanisms underlying neurodegenerative disease. For example, in AD the basal forebrain cholinergic nuclei are more affected than the midpontine cholinergic nuclei in the brainstem, whereas the reverse is seen in Parkinson's disease (Pepeu and Grazia Giovannini, 2017). Whilst one previous study has shown reduced basal forebrain volumes in patients with both CBD and PSP (Kasashima and Oda, 2003), our results demonstrated significant differences only in cases with PSP pathology. This is in line with other research that has shown relative preservation of basal forebrain nuclei in CBD compared to PSP (Dickson, 1999). Taken together, this research suggests there is a greater vulnerability of the basal forebrain cholinergic nuclei to certain forms of tau pathology. Importantly, it has been suggested previously that tau only causes neurodegeneration in the basal forebrain in tauopathies where there is also concurrent amyloid deposition, for example in AD (Pepeu and Grazia Giovannini, 2017). However, our research has shown this is not the case, as individuals with primary tauopathies do not have co-occurring amyloid pathology.

We also show that individuals with TDP-43 type C pathology had significantly smaller basal forebrain volumes than controls. This is consistent with the finding in the clinical groups of decreased volume in svPPA, as the majority of patients with this phenotype have TDP-43 type C pathology (Bergeron et al., 2018). TDP-43 pathology is seen in the basal forebrain in amyotrophic lateral sclerosis (Cykowski et al., 2014), however the role of TDP-43 in the basal forebrain in FTD has not been widely investigated. One study has shown the greatest severity of TDP-43 inclusions in the whole brain were in the basal forebrain (Cykowski et al., 2016). Furthermore, within this population the most prominent TDP-43 pathology was seen in the diagonal band of Broca in a subject with FTD (Cykowski et al., 2016). Therefore, there is some evidence to suggest that the basal forebrain is vulnerable to TDP-43 pathology in FTD patients, although more research into the type of TDP-43 pathology present in the basal forebrain in distinct clinical subtypes of FTD is required.

The brain cholinergic system is an extensive network of projection neurons that innervate several brain areas. Neurons arise from the peduncolo-pontine nucleus (PPN) and dorsolateral tegmental nucleus (DLN) in the brainstem and project to the thalamus, hypothalamus, globus pallidus, striatum, and the basal forebrain (Dautan et al., 2014; Hallanger and Wainer, 1988; Pepeu and Grazia Giovannini, 2017). Subcortical atrophy in FTD has previously been demonstrated in the thalamus, (Bocchetta et al., 2018; Rohrer et al., 2010), hypothalamus (Bocchetta et al., 2015), and striatum (Chow et al., 2008). These regions are highly connected by cholinergic neurons to the basal forebrain, therefore it is perhaps unsurprising that this region is similarly affected. One study has shown that hypometabolism in the septal region of the basal forebrain is associated with reduced performance on a prosocial sentiment task in individuals with bvFTD (Moll et al., 2011), suggesting this region may play a role in social cognition. Furthermore, other research has suggested the basal forebrain is important for language function (Simić et al., 1999; Teipel et al., 2016, 2014), which is of particular interest considering individuals with svPPA had significantly reduced volumes in this study. Due to the extensive cholinergic network involved, it is likely that basal forebrain volume reductions play a prominent role in cognitive impairment and language function in FTD. However, more research is needed to investigate the role of the basal forebrain and resulting impairment in FTD.

Whether atrophy of the basal forebrain disrupts the entire cholinergic system in FTD remains unclear. However, there is no evidence that anticholinesterase therapies are helpful in bvFTD or PPA (Noufi et al., 2019), and in fact some evidence that cholinesterase inhibitors can worsen behaviour in bvFTD (Kertesz et al., 2008; Mendez et al., 2007). However, trials will have included patients with multiple different types of FTD pathology, and the work here suggests that such therapy may be helpful in specific subsets of patients.

To our knowledge, this is the first study to extensively investigate the role of the basal forebrain in FTD, particularly in those with confirmed genetic and pathological forms. This was a retrospective study, and as such, accompanying clinical and neuropsychological data were not uniformly collected and therefore not available for comparative analysis. Furthermore, a number of our patient groups had small sample sizes, and therefore results should be interpreted with caution, and replication in larger datasets will be important. It will also be important to use higher resolution MR imaging to better understand the differential involvement of specific basal forebrain subnuclei within the different clinical, genetic, and pathological subtypes of FTD.

## CRediT authorship contribution statement

**Rhian S. Convery:** Writing - original draft, Formal analysis, Writing - review & editing. **Mollie R. Neason:** Data curation. **David M. Cash:** Data curation. **M. Jorge Cardoso:** Data curation. **Marc Modat:** Data curation. **Sebastien Ourselin:** Data curation. **Jason D. Warren:** Data curation. **Jonathan D. Rohrer:** Conceptualization, Data curation. **Martina Bocchetta:** Conceptualization, Data curation.

## Appendix Supplementary materials

Image, application 1
