# Supplementary material for: Basal forebrain atrophy in frontotemporal dementia
Source: Neuroimage Clin. 2020 Feb 13;26:102210. doi: 10.1016/j.nicl.2020.102210 (PMC7058403; doi:10.1016/j.nicl.2020.102210)
Supplement: Supplementary file 1 [file mmc1.docx]

**Supplementary Material**

**Queen Square Brian Bank protocol**

All patients consented to brain donation at the Queen Square Brain Bank for Neurological Disorders (QSBB). After death the brains were processed according to QSBB protocol, which involves freezing of the right brain hemisphere, and fixation in 10% buffered formalin with tissue sampling and processing for paraffin histology of the left hemisphere. All tissues are stored at QSBB under a licence from the Human Tissue Authority. Sections of 7 μm thickness were cut from formalin-fixed paraffin-embedded tissue blocks from multiple brain regions, mounted on glass slides and stained with haematoxylin and eosin (H&E). Representative sections were examined with immunohistochemical stains with the following antibodies: phospho-tau (AT8 MN1020, 1:600; Thermo), Aβ (6F3D, 1:100; Dako), α-synuclein (ab80627, 1:1500; Abcam), TDP-43 (2E2-D3, 1:5000; Abnova), 4R tau (1E1/A6, 1:4000; Millipore) and 3R tau (8E6/C11, 1:800; Millipore). All immunostainings were carried out on an automated immunostaining machine (A.Menarini Diagnostics) following manufacturer’s guidelines using 3,3′-diaminobenzidine as chromogen and appropriate positive and negative controls. Ethical approval for the study was obtained from the Local Research Ethics Committee of the National Hospital for Neurology and Neurosurgery.

**Supplementary Table 1. Individual mutations for *GRN* and *MAPT* included in the study. Numbers in brackets denote the number of patients carrying that mutation included in the study.**

| **Mutations** | |
| --- | --- |
| ***GRN (number)*** | ***MAPT (number)*** |
| M1 (2T>C) *(1)* | D252V *(1)* |
| C31fs *(6)* | G271R *(1)* |
| Q130fs *(2)* | P301S *(1)* |
| S203fs *(2)* | 10+14 *(1)* |
| IVS7+5_8delGTGA *(1)* | 10+16 *(15)* |
| A350fs *(1)* | S320F *(1)* |
| C482X *(1)* | Q351R *(2)* |
| E498fs *(1)* | R406W *(2)* |
| **Total *(15)*** | **Total *(24)*** |
